# Growth hormone retesting for idiopathic isolated growth hormone deficiency during and after puberty: a systematic review

**DOI:** 10.1210/jendso/bvag092

**Published:** 2026-04-10

**Authors:** Joeri Vliegenthart, Deveney F Wols, Jan M Wit, Wichor Bramer, Edmond H H M Rings, Erica L T van den Akker, Danielle C M van der Kaay

**Affiliations:** Division of Pediatric Endocrinology, Erasmus MC Sophia Children Hospital: Erasmus MC Sophia Kinderziekenhuis, 3000 CA Rotterdam, The Netherlands; Division of Pediatric Endocrinology, Erasmus MC Sophia Children Hospital: Erasmus MC Sophia Kinderziekenhuis, 3000 CA Rotterdam, The Netherlands; Division of Pediatric Endocrinology, WAKZ: Leids Universitair Medisch Centrum Willem Alexander Kinderziekenhuis, 2300 RC Leiden, The Netherlands; Medical Library, Erasmus Medical Centre: Erasmus MC, 3000 CA Rotterdam, The Netherlands; Department of Pediatrics, Erasmus MC Sophia Children Hospital: Erasmus MC Sophia Kinderziekenhuis, 3000 CA Rotterdam, The Netherlands; Division of Pediatric Endocrinology, Erasmus MC Sophia Children Hospital: Erasmus MC Sophia Kinderziekenhuis, 3000 CA Rotterdam, The Netherlands; Division of Pediatric Endocrinology, Erasmus MC Sophia Children Hospital: Erasmus MC Sophia Kinderziekenhuis, 3000 CA Rotterdam, The Netherlands

**Keywords:** pituitary dwarfism, human growth hormone, gonadal steroid hormones, pituitary function tests

## Abstract

**Context:**

Idiopathic isolated growth hormone deficiency (IIGHD) is difficult to diagnose due to the day-to-day variation in growth hormone (GH) secretion and limitations of GH stimulation testing (GHST). Although recombinant human GH (rhGH) is typically continued until near adult height (NAH), many patients no longer show deficiency at that point. The optimal timing and method for retesting GH secretion remain unclear.

**Objective:**

To summarize the currently available GHSTs in children with IIGHD, with particular focus on diagnostic strategies in the peripubertal period, timing of retesting, and the role of sex steroid priming.

**Methods:**

A systematic literature search was conducted in 4 databases up to June 2025. Studies were included if they reported on GH retesting in children with IIGHD. Data were extracted on patient characteristics, GHST protocols, priming strategies, cutoff values, and reversal rates. Risk of bias was assessed using the ROBINS-I tool.

**Results:**

Thirty-one studies involving 2057 patients were included. Retesting occurred after 1–2 years of rhGH treatment, during mid-puberty, or at (N)AH, with mean reversal rates of 46.4%, 46.3%, and 69.6%, respectively. Priming with sex steroids was inconsistently applied, using testosterone in boys and ethinyl estradiol in girls. A GH peak cutoff of 7 μg/L was most commonly used, though values varied. Mid-pubertal retesting may reduce false-positive diagnoses and treatment burden.

**Conclusion:**

Retesting strategies for IIGHD should be individualized based on treatment response and pubertal development. Early retesting is advised for poor responders, while mid-puberty retesting suits most patients with normal development. Priming is recommended in older prepubertal children. Standardization of GHST protocols and long-term outcome studies are needed to optimize care and reduce overtreatment.

Growth hormone deficiency (GHD) is a rare cause of short stature with a recent epidemiological study reporting a prevalence ranging from 1:1100 to 1:8600 children [1]. An accurate diagnosis is important, considering the significant impact that recombinant human growth hormone (rhGH) treatment can have on a patient's health. Differentiating GHD from idiopathic short stature; including constitutional delay of growth and puberty (CDGP) remains a clinical challenge—particularly in children with idiopathic isolated GHD (IIGHD), defined as clinical and/or laboratory features of GHD without structural abnormalities of the hypothalamic-pituitary region or genetic defects [2]. Since growth hormone (GH) secretion is pulsatile and random GH measurements are not informative, growth hormone stimulation testing (GHST) is currently considered the diagnostic gold standard for identifying GHD [3]. In this test, pharmacological agents are used to provoke GH release, and failure to reach a defined GH peak supports the diagnosis of GHD.

Over 34 provocative tests have been developed [4]. Currently, a variation of provocative GH tests is used. In children, the most frequent pharmacological agents used are clonidine, arginine, glucagon and insulin [4, 5].

Interpretation of the results of GHST must be done carefully due to significant variation in its reproducibility as well as the high percentage of false-positive tests [6]. Even though tests are performed at least twice in children with IIGHD, results can differ widely, complicating interpretation and diagnostic accuracy. Additionally, cutoff values have not been determined through scientific evidence but reached by consensus [7]. Factors such as pubertal status leading to an increase in GH secretion during mid- and late puberty in response to rising levels of sex steroids, and body mass index (higher BMI is associated with lower GHST cutoffs) affect the outcome [8‐12]. In contrast, during the initial phase of puberty in males, physiological 24-hour GH concentrations are low, which increases the risk of false-positive results in GH stimulation tests [9, 10, 13, 14].

There is no consensus among pediatric endocrinologists on the role of priming with sex steroid hormones in prepubertal children or those in the initial stages of puberty prior to provocative GH testing [15, 16]. Priming with sex steroids increases GH secretion, thereby potentially eliminating false-positive cases due to subnormal secretion caused by a lack of sex hormones rather than by GHD [17‐20]. Omission of priming before testing could be a source of error in falsely diagnosing GHD due to a decrease in specificity [18, 21].

After initiating rhGH treatment, it is advisable to consider retesting GH secretion at an appropriate point during follow-up in patients with IIGHD. Such strategy is essentially a repetition of the diagnostic approach but has the advantage that information will be available on the short-term growth response to rhGH treatment. Another advantage is that it will identify children with a false-positive test result at initial testing and avoid unnecessary continuation of treatment [22‐25]. An alternative approach is to retest during mid-puberty, when physiological exposure to sex steroids would theoretically reduce the likelihood of false-positive GHST results [25, 26]. This strategy may enable earlier discontinuation of treatment. The current standard of retesting at near adult height (NAH) is primarily intended to guide the transition to adult care [27‐29]. Retesting is performed after stopping rhGH treatment. A sufficient IGF-1 level at this point increases the likelihood of transient rather than persistent GHD [7, 30‐32]. However, this strategy is associated with the greatest treatment burden and carries a significant risk of prolonged treatment in patients who may no longer meet the criteria for GHD.

Firstly, this systematic review aims to provide an overview of the currently available growth hormone stimulation tests and the preferred diagnostic tool for peripubertal children with idiopathic isolated GH deficient. Secondly, we discuss timing of retesting and the use of priming with sex steroids before testing.

## Materials and methods

### Eligibility criteria

Studies were screened for eligibility criteria. Studies must have included (sub)groups of patients with IIGHD and patients were treated with rhGH. Growth hormone stimulation testing must have been performed, in puberty or at any other time.

### Information sources and search strategy

The search strategy was developed in cooperation with the staff of the Erasmus MC Medical Library. The search included various years of coverage, depending on the database creation. The following databases were searched for publications: Embase (1971–10th June 2025) via Embase.com, Medline ALL (1946–10th June 2025) via Ovid, Web of Science Core Collection (1975–10th June 2025) via Web of Knowledge, and Cochrane Central Register of Controlled Trials (1992–10th June 2025) via Wiley.

The complete search strategies are provided in Supplementary File 1 [33]. The search combined terms for (1) GHD or pituitary dwarfism; (2) puberty or pediatrics; (3) provocative or stimulation test for diagnosis. Searches were limited to English and excluded animal studies. After deduplication in EndNote [34], articles were screened for eligibility. Reference lists of included studies and relevant reviews were hand-searched. Abstracts and titles were screened for inclusion criteria for this review. The PRISMA statement was used as guidance to report this systematic review [35, 36]. Two reviewers (D.W. and J.V.) independently assessed eligibility in a standardized, unblinded manner, with discrepancies resolved by discussion and consensus.

### Data collection process

Study characteristics were extracted using a standardized Excel sheet by 2 independent reviewers (D.W. and J.V.), who cross-checked each other's data.

### Data items

Data were extracted from each included study on the following patient characteristics: age, sex, pubertal stage, diagnostic measurements (priming status, initial diagnosis method, and cutoff values), and timing of retesting and method (including cutoff values and GH withdrawal duration before retesting). We also assessed GHD reversal rate, defined as the proportion of patients who no longer met the criteria for GHD upon retesting. To use the same unit for cutoff values, reported GH levels in mU/L were divided by 3 to convert to micrograms per liter (µg/L).

### Risk of bias in individual studies

To assess the risk of bias in individual studies, the ROBINS-I tool was used [37]. Two reviewers (J.V. and D.W.) independently evaluated the ROBINS-I components, including bias due to confounding, selection of participants, classification of interventions, deviations from intended interventions, missing data, measurement of outcomes, and selection of the reported result. This information was used to determine the reliability of study results. The robvis tool was used to visualize the risk of bias assessments [38].

### Summary measures

The principal summary measures used in the analysis include the mean age at diagnosis and retesting, the frequency of various GHSTs that were used, the GH cutoff levels at diagnosis and retesting, the use of sex steroid priming, the duration of rhGH withdrawal before retesting, and the reversal rates at retesting.

### Synthesis of results

Microsoft Excel (Version 16.92) was utilized to construct the characteristics table. The data were analyzed using IBM SPSS Statistics (Version 29.0.1.0). For each study, the 95% confidence interval (CI) for the reversal rate was calculated using the Wald method for binomial proportions. The reversal rate was defined as the number of patients with reversal divided by the total number of retested patients. The standard error (SE) of the proportion was calculated using the formula: SE = square root of (*P* × (1 − *P*) ÷ *n*), where *P* is the observed proportion (reversal rate) and *n* is the number of patients retested. The 95% CI was then calculated as: CI = *P* − 1.96 × SE to *P* + 1.96 × SE. Confidence intervals were expressed as percentages. For summary estimates per retest moment, reversal rates and sample sizes were aggregated across studies, and the same method was applied.

## Results

### Study selection

**Figure 1:**
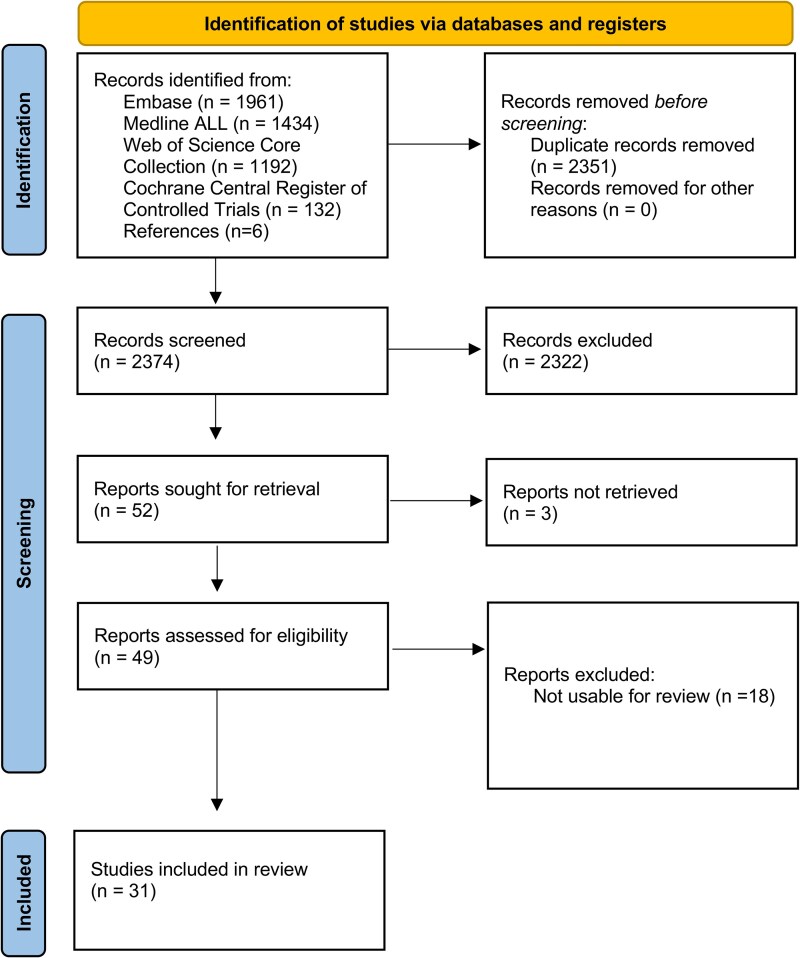
Flowchart illustrating the study selection process based on the PRISMA 2020 guidelines [36].

A total of 2374 studies were screened, 2322 were excluded in the initial phase due to irrelevance. Out of the 52 studies sought, 3 were not found. The remaining 49 studies were assessed for eligibility, 18 did not meet the eligibility criteria. This left a total of 31 studies, as shown in Fig. 1.

### Study characteristics

All included studies (*n* = 31) are shown in Table 1. The publication years ranged from 1987 to 2024.

**Table 1:** Summary of study characteristics and outcomes

Author	Year	IGDH patients	Total *n* =	Age diagnosis	M	F	Priming before initial test	Type initial test	Cutoff initial test	Type retest	Priming before retest	Time of withdrawal before retest	Pubertal stage	Moment of retest	Age at retest	Cutoff retest	% GH sufficient after retest
Ahmid [39]	2016	31	74	10.7	78	52	?	1 GHST	6.6 ug/L	1 GHST and/or serum IGF-1	N/A	6 months	AH	NAH	18.2	5 μg/L	29.0%
Aimaretti [40]	2000	23	62	?	15	8	No	2 GHSTs	10 µg/L	GHRH + Arg, ITT on indication	N/A	>3 months	AH	NAH	23.0 ± 1.5	GHRH + Arg: 16.5 μg/L*^a^* ITT: 5.0 μg/L*^a^*	34.8%
Berberoglu [41]	2008	52	70	?	29	23	No	2 GHSTs	10 μg/L*^a^*	ITT	N/A	6 weeks	AH	NAH	16.8 ± 2.0	3 μg/L*^a^*	82.7%
Bizzarri [22]	2015	38	38	8.78 ± 2.4	22	16	2 patients	2 GHSTs: CST + AST, GST, ITT, GHRH + Arg	GHRH + Arg: 20 μg/L*^a^*, Other: 8 μg/L*^a^*	GHRH + Arg	3 patients	9 weeks	14 prepubertal/12 mid-puberty/12 pubertal	>1 year after starting treatment	?	20 μg/L*^a^*	94.7%
Bonfig [42]	2008	77	90	9.7 ± 3.7	68	22	Yes	2 GHSTs: CST, AST, ITT	10 μg/L*^a^*	ITT	N/A	1 year	AH	NAH	17.8 ± 1.8	5 μg/L	75.3%
Cacciari [43]	1994	184	184	9.6 ± 2.8	113	71	?	AST, L-Dopa, Sleeptest	8 μg/L	AST, L-Dopa, Sleep test	No	4 weeks	First retesting: 140 prepubertal/44 pubertal. Second retesting: 57 prepubertal/11 pubertal	3 y + 4.5 y after starting treatment	?	8 μg/L	18.5%
Cacciari [24]	1992	63	63	10.2 ± 3.2	38	25	No	AST, L-Dopa, Sleeptest	8 μg/L	AST, L-Dopa, Sleep test	No	4 weeks	28 prepubertal, 12 pubertal, 23 pubertal before treatment	2 years after starting treatment	?	8 μg/L	33.0%
Cavarzere [26]	2020	80	80	9.9 (M); 9.6 (F)	46	34	No	2 GHSTs: AST, ITT, GST	8 μg/L	AST	N/A	12 weeks	Mid-puberty	Mid-puberty	11.4 (M); 12.2 (F)	8 μg/L	55.0%
Clayton [21]	1987	19	40	11.8	26	14	Yes	2 GHSTs: ITT, AST, Bovril, GST, CST	5 μg/L***^b^***	ITT, AST	N/A	6 months	AH	NAH	16.0-19.2	5 μg/L*^b^*	26.3%
Darendeliler [31]	2004	30	50	7.9 ± 3.3	33	17	No	ITT, L-Dopa	10 μg/L*^a^*	ITT, L-Dopa	Yes	?	Different stages	Variable	15.2 ± 5.0	10 μg/L*^a^*	43.0%
Deillon [44]	2015	30	50	9.5 ± 3.6	42	21	Yes	2 GHSTs: GST, AST, ITT	10 μg/L	AST, ITT + Arg, ITT	N/A	1-4 months	AH	NAH	17.8 ± 1.8	10 μg/L	73.3%
Dreismann [45]	2016	126	149	7.1 ± 3.2	108	41	3 patients	2 GHSTs: AST, ITT, CST, Night	8-10 μg/L*^a^*	GHRH + Arg	N/A	3 months	NAH	NAH	16.8 ± 1.7	15.9 μg/L	81.9%
Gelwane [46]	2007	24	62	9.8 ± 2.5	15	9	No	ITT + Arg	10 μg/L	ITT, GST + propranolol	N/A	8 months	AH	NAH	16.8 ± 1.6	10 μg/L	62.5%
Goksen [47]	2001	6	9	12.8 ± 2.6	7	2	Yes	ITT, L-Dopa	2.3 μg/L*^b^*	ITT, L-Dopa	N/A	4.6 years	AH	NAH	21.0 ± 3.0	2.3 μg/L*^b^*	16.7%
Juul [7]	1997	62	1345	12.1 ± 0.7	73	35	?	CST	?	CST	N/A	>6 months	AH	NAH	22.2 ± 0.7	7.5 μg/L	45.2%
Longobardi [48]	1996	54	107	?	40	29	?	2 GHSTs: AST, ITT, L-Dopa	?	GHRH + PD & ITT	N/A	?	AH	NAH	25.4 ± 5.8	10 μg/L	46.3%
Maghnie [49]	1999	23	35	9.0 ± 2.5	15	8	?	?	10 μg/L	AST, ITT, on indication ITT + Arg	N/A	3 months	AH	NAH	19.2 ± 3.2	10 μg/L	81%
Maghnie [50]	2005	8	26	?	17	9	?	2 GHSTs	10 μg/L	ITT	N/A	3 months	AH	NAH	20.8 ± 2.3	5 and 3 μg/L	12.5% or 37.5%
Meazza [28]	2017	163	164	10.4 ± 3.6	109	55	?	AST, GST	10 μg/L*^a^*	GHRH + Arg or AST	N/A	6-8 weeks	AH	NAH	16.2 ± 1.4	GHRH + Arg: 19 μg/L*^a^*, AST 10 μg/L*^a^*	82.8%
Nicolson [51]	1996	32	88	11.1	49	39	?	1/2 GHSTs: ITT, AST, GST, CST, Bovril	6.6 μg/L*^b^*	AST and/or ITT	N/A	<2 year	AH	NAH	17.5 (10.8-33.5)	3 μg/L*^b^*	46.9%
Pauwels [52]	1992	15	15	8.2 ± 0.7	10	5	?	AST, ITT	8 μg/L	AST, ITT	N/A	1.4 year	AH	NAH	17.8 ± 0.3	8 μg/L	53.3%
Penta [53]	2019	31	31	10.7 ± 2.9	19	12	No	2 GHSTs: CST, AST, GHRH + Arg	CST/AST: 8 μg/L*^a^* GHRH + Arg 20 μg/L*^a^*	GHRH + Arg	No	>3 months	AH	NAH	15.95 ± 1.20	19 μg/L*^a^*	83.9%
Quigley [54]	2013	41	73	10.5 ± 3.3	24	17	?	2 GHSTs	5 μg/L	AST/ L-Dopa, AST, ITT, ITT + Arg, ITT/CST, ITT/ L-Dopa, L-Dopa	N/A	6 weeks – 5y	AH	NAH	17.6 ± 1.8	5 μg/L	82.9%
Smyczynska [55]	2014	150	150	12.5 ± 2.7	117	33	?	CST, ITT, GST	10 μg/L	CST, ITT	N/A	1 month	AH	NAH	17.3 ± 1.1	6 μg/L	88.0%
Smyczynska [56]	2024	260	260	13.2 (11.9;14.4)	173	87	No	CST, GST	7.0 µg/L	ITT, CST	N/A	1-6 months	AH	NAH	17.6(16.5;18.3)	10.0 µg/L	64.4%
Tauber [29]	1997	121	131	12.9 ± 2.8	79	52	Yes	2 GHSTs: ornithine, L-Dopa, CST, AST, GST, CST + betaxolol, ITT + Arg, GST + propanolol, GST + betaxolol	10 μg/L	CST, betaxolol	?	15 days	AH	NAH	16.7 ± 1.7	5 μg/L	66.9%
Thomas [57]	2003	33	43	10.7 ± 3.0	23	20	Yes	GST, ITT	10 μg/L*^a^*	GST and/or ITT	Yes	2-6 weeks	Prepubertal 33, pubertal 10	Multiple times	17.3 ± 1.5	3 and 10 μg/L*^a^*	36.4%
Vuralli [23]	2017	170	265	10.6 ± 3.5	161	104	Yes	CST, L-Dopa	10 μg/L*^a^*	CST	Yes	1 week	69 prepubertal IGHD patients at retest, others pubertal	1y after starting treatment	11.2 ± 3.1	10 μg/L*^a^*	40.6%
Wacharasindhu [58]	1996	8	8	10.8 ± 2.0	7	1	Yes	ITT	3 μg/L*^b^*	ITT	N/A	3 months	AH	NAH	17.4 ± 1.4	5 μg/L*^b^*	87.5%
Wacharasindhu [59]	2015	14	24	8.9 ± 2.9	9	5	?	ITT, CST	10 μg/L*^a^*	ITT	N/A	?	AH	NAH	17.6 ± 2.9	5 μg/L*^a^*	64.3%
Zucchini [25]	2006	69	69	9.4 ± 1.9	40	29	No	AST, L-Dopa	10 μg/L	AST, L-Dopa, Sleep test	N/A	4-6 weeks	Mid-puberty	>2y after starting treatment	13.0 ± 1.3	10 μg/L	36.2%

Abbreviations: AH, adult height; AST, arginine stimulation test; CST, clonidine stimulation test; GHRH, growth hormone-releasing hormone; GHRH + Arg, growth hormone-releasing hormone with arginine; GHST, growth hormone stimulation test; GST, glucagon stimulation test; IGF-1; insulin-like growth factor 1; IGHD, isolated growth hormone deficiency; ITT, insulin tolerance test; L-Dopa, levodopa; MPHD, multiple pituitary hormone deficiencies; NAH, near adult height; ?, unknown.

^*a*^Original study reported in ng/mL.

^*b*^Original study reported in mU/L.

In these studies, a total of 3855 patients (including 2057 patients with IIGHD) were included. Study size ranged from 8 to 1354 for all patients within a single study, and from 6 to 260 for patients with IIGHD.

### Results of individual studies

The age at diagnosis for patients with IIGHD ranged from 7.1 to 13.2 years and was not reported by 4 studies. The age at retesting varied from 11.2 to 25.4 years, with 5 studies focusing on patients below the age of 16 years.

#### GHST at GHD diagnosis

GHSTs used at diagnosis included the insulin tolerance test (ITT) (*n* = 20), arginine stimulation test (AST) (*n* = 15), clonidine stimulation test (CST) (*n* = 13), glucagon stimulation test (GST) (*n* = 11), L-Dopa stimulation test (*n* = 9), sleep test (*n* = 3), growth hormone-releasing hormone + AST (GHRH + Arg) (*n* = 3), bovril stimulation test (*n* = 2), ornithine stimulation test (*n* = 1), propranolol stimulation test (*n* = 1), betaxolol stimulation test (*n* = 1), and spontaneous night secretion of GH (night) (*n* = 1). The GH cutoff levels at diagnosis were between 2.3 and 10 μg/L, with an exception for GHRH + Arg, for which higher cutoffs are used (20 μg/L in 2 studies) due to its distinct physiological mechanism and stronger stimulation of GH secretion [22, 53]. The most common cutoff level was 10 μg/L, occurring 15 times. Two studies did not report the initial cutoff level at diagnosis [7, 48].

#### Retesting for GHD

GHSTs used for retesting included ITT (*n* = 22), AST (*n* = 10), L-dopa (*n* = 7), CST (*n* = 6), GHRH + Arg (*n* = 5), sleep test (*n* = 3), GST (*n* = 2), propranolol (*n* = 1), and betaxolol (*n* = 1).

Retesting was performed 22 times at NAH or upon completion of growth. In the other studies, retesting was conducted after a specific duration from start of rhGH treatment. Vuralli et al and Thomas et al retested after 1 year of treatment, 2 patients in Thomas's group underwent a second retest 2 years after the first [23, 57]. Cacciari et al retested 1.8 years after starting rhGH treatment and again 1.5 years later [24, 43].

In 2 studies variable moments for retesting were used [22, 31]. Darendeliler et al included 10 prepubertal patients, while Bizzarri et al included 14 prepubertal patients and 24 patients at various stages of puberty (12 with Tanner stage 2-3 and 12 with Tanner stage 4-5) [22, 31]. Zucchini et al and Cavarzere et al retested in mid-puberty [25, 26]. Zucchini et al retested males with a testicular volume (TV) of 6-12 mL and females at Tanner stage B2-3, while Cavarzere et al retested males at Tanner stage 3 with an TV ≥10 mL, and females at Tanner B3 [25, 26]. In the study of Cacciari et al [24], 28 subjects were prepubertal at the time of retesting, 12 had started puberty (Tanner stage 2 or higher), and 23 were pubertal before start of rhGH treatment.

**Figure 2:**
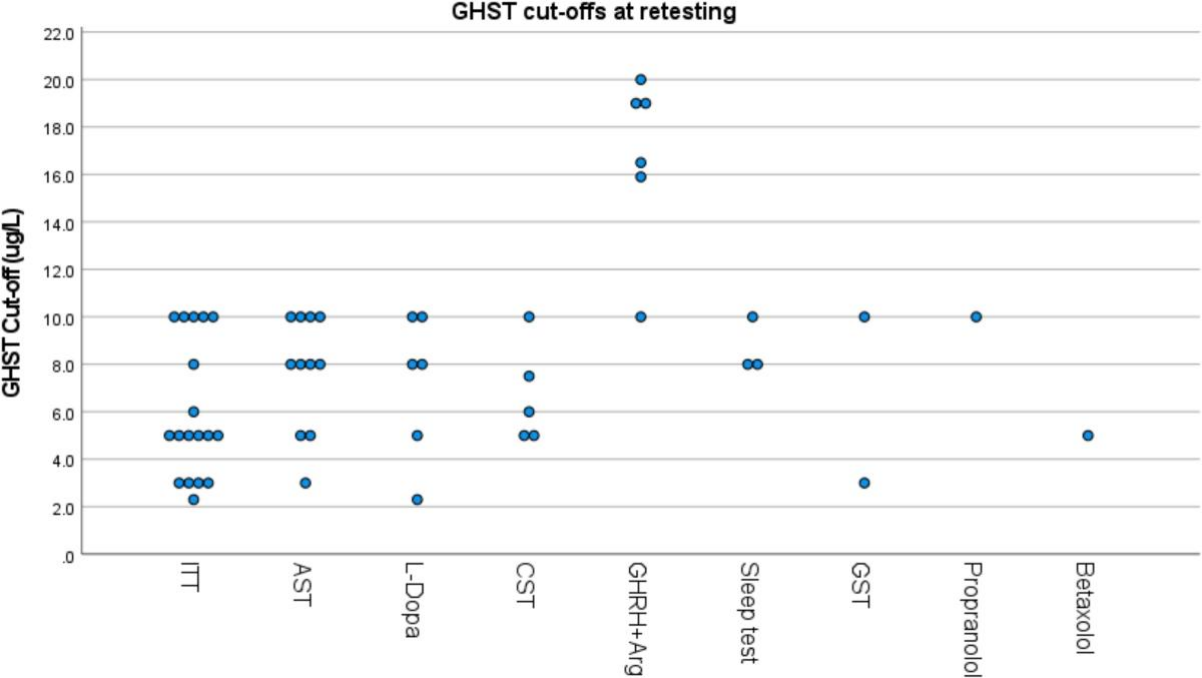
Distribution of GH cutoff levels used at retesting across included studies. Cutoff values ranged from 2.3 to 20 μg/L. Four studies applied multiple cutoff levels depending on the GH stimulation test (GHST) protocol. AST, arginine stimulation test; CST, clonidine stimulation test; GHRH + Arg, growth hormone-releasing hormone + arginine; GST, glucagon stimulation test; ITT, insulin tolerance test; L-Dopa, levodopa stimulation test.

As shown in Fig. 2, the GH cutoff levels at retesting were 2.3-20 μg/L. The most common cutoff levels were 10 μg/L (9 times) and 8 μg/L (6 times). Four studies used more than 1 cutoff at retesting, depending on the GHST used [28, 40, 50, 57].

The duration of rhGH withdrawal before retesting varied widely, ranging from 1 week to 5 years, with the most common intervals being 3 months (*n* = 6), 4 weeks (*n* = 4), and 6 months (*n* = 3). Three studies did not report the time of rhGH withdrawal before retesting [31, 48, 59].

#### Priming before GHST

Sex steroid priming before the GHST was used in 10 studies at diagnosis, while in 12 studies its use was not reported. Four studies used sex steroid priming at retesting [22, 23, 31, 57]. Two studies included prepubertal children at retesting, but did not use priming [24, 43]. All other studies included patients at mid-puberty or NAH when sex steroid priming is not required. Criteria for priming were: prepubertal at testing and an age for boys older than 9 (*n* = 1), 10 (*n* = 2), or 12 (*n* = 2), and for girls 9 (*n* = 2), 10 (*n* = 3), or 12 (*n* = 1). For boys, the dosage of intramuscular testosterone varied between 50, 100, or 125 mg testosterone 3 days before testing or 100 mg testosterone 1 week before testing (*n* = 2). Prepubertal girls were primed with various dosages and schedules of ethinyl estradiol: 50 or 120 μg/day for 3 or 7 days before the test, or 1 mg Stilbestrol for 2 days before the test [21‐23, 29, 31, 42, 44, 45, 47, 57, 58].

#### Reversal rate after retesting

**Figure 3:**
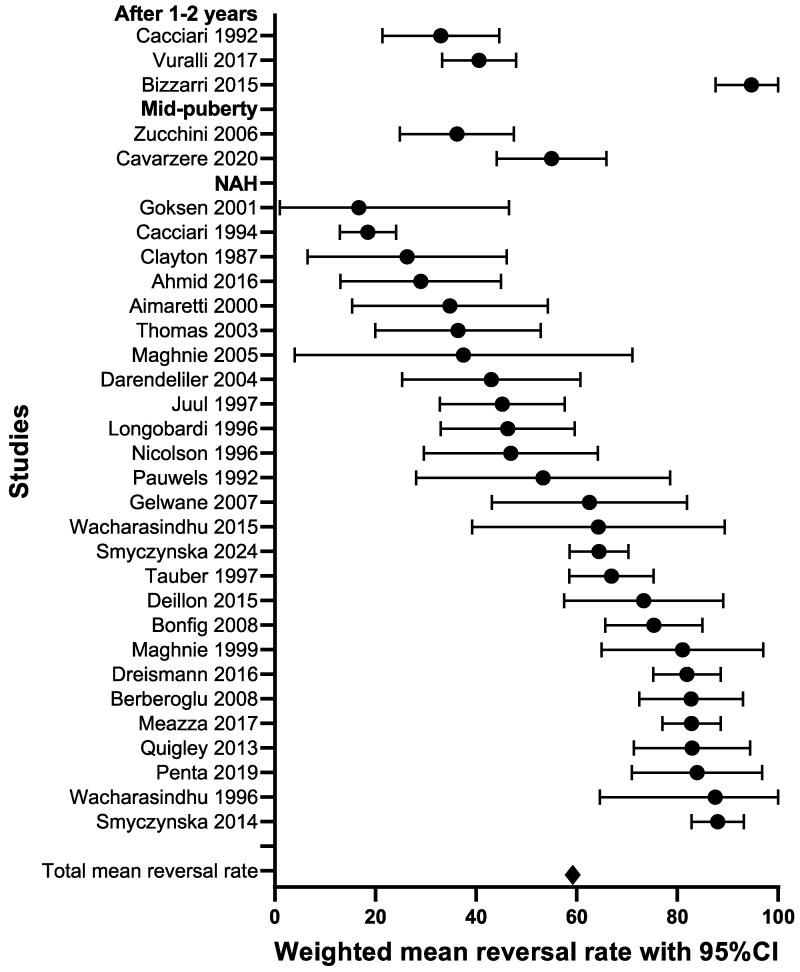
Forest plot showing individual study reversal rates and the overall weighted mean reversal rate with 95% CI. Each horizontal line represents the 95% CI for the reversal rate reported in a study, while the diamond at the bottom indicates the pooled estimate across all included studies. The studies are grouped by timing of retesting: after 1-2 years, during mid-puberty, and at NAH.

**Figure 4:**
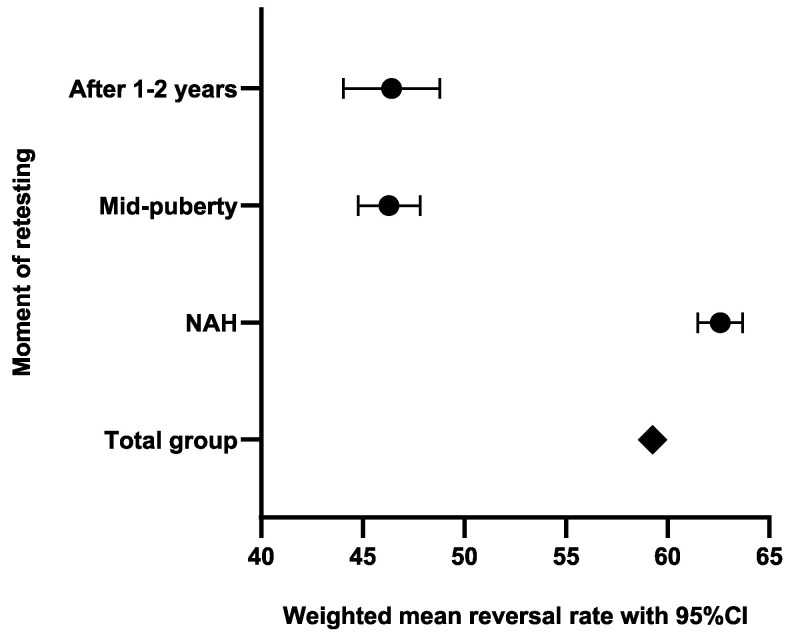
Forest plot showing the weighted mean reversal rate with 95% CIs for 3 distinct time points of retesting: after 1-2 years, at mid-puberty, and at near adult height (NAH). The bottom diamond represents the overall mean reversal rate across all groups.

The reversal rate after retesting varied from 12.5% to 100%. The study that reported a reversal rate of 100% did so specifically for 1 of the GHSTs used (ITT + Arg: 100%, AST: 61.1%, ITT: 77.8%) [49]. To account for differences in study sample sizes, a weighted mean reversal rate was calculated across the 31 included studies. The weighted mean (SD) reversal rate was 58.7% (22.9%), as shown in Fig. 3. When we only selected the studies that retested before AH was reached, a weighted mean reversal rate of 36.9% (18.1%) was found in patients ranging in age from 9.7 to 15.2 years [22‐26, 31, 43, 57]. In contrast, when considering only the studies conducted at NAH/AH the weighted mean reversal rate was 69.6% (16.3%), with an age range of 16.0-25.4 years. The weighted mean reversal rate in studies that retested 1-2 years after IIGHD diagnosis was 46.4% (16.8%) [22‐25]. A weighted mean reversal rate of 46.3% (9.4%) was found in the studies that investigated reversal rate at mid-puberty [25, 26]. Figure 4 presents the weighted mean reversal rates with corresponding 95% CI for each retesting moment.

### Risk of bias within studies

Based on the ROBINS-I assessment, risk of bias among varied across studies: 9 were rated as low risk of bias, 19 as moderate risk, and 3 studies were judged to have serious risk of bias. A visualization is provided in Supplementary Files 2 and 3 [33]. The studies with serious risk of bias were primarily affected by confounding and missing data, with critical issues in the adjustment for confounders and handling of incomplete datasets [48, 54]. Studies rated as moderate risk demonstrated generally low risk of bias across most domains, with moderate risk arising from isolated challenges, such as confounding, selection of participants, or missing data. Notably, the studies classified as low risk demonstrated consistent adherence to robust methodological standards, including detailed reporting of outcomes. In 12 out of 30 studies, it was unclear whether sex steroid priming was performed in prepubertal children at diagnosis, 4 studies failed to report age at diagnosis, and 6 studies did not specify the GHST used at diagnosis.

## Discussion

### Summary of evidence

In this review, we analyzed all studies conducted since 1987 that have examined retesting of patients with GHD at various time points after the initiation of rhGH treatment, with a special focus on retesting after 1-2 years and at mid-puberty.

#### How to retest?

When considering retesting for GHD, most studies in this review, of which both the diagnostic test and the retest are known, have used the same test at both times to ensure consistency, except for 5 studies. During puberty, the most commonly used tests for retesting include ITT, AST, L-Dopa, CST, and GHRH + Arg. The ITT is validated for diagnosing adult GHD and persistent GHD during the transition period [27]. Since the end of 2022, GHRH is unavailable for clinical use worldwide. Centers that were used to perform the GHRH + Arg test are exploring alternative diagnostic methods [60, 61].

#### When to retest?

##### Retesting after 1 year of rhGH treatment

Retesting 1 year after starting rhGH treatment offers several advantages. It allows the growth response to serve as an additional criterion to verify that low GH secretion was the cause of poor growth. This approach is easier to explain to patients and their parents and results in lower rhGH waste and reduced patient burden compared with retesting at NAH. However, a disadvantage is the uncertain cutoff for a poor growth response. In this review, the mean reversal rate of studies that retested 1-2 years after start of rhGH treatment is approximately 47%, indicating that retesting at this point can be worthwhile [22‐25].

##### Retesting at mid-puberty

A mean reversal rate of 46.3% was found in the studies that investigated reversal rate at mid-puberty [25, 26]. One of the main advantages is the benefit of relatively long exposure to physiological sex steroids. This probably decreases the percentage of false-positive GHST results, although for males this only applies to mid-puberty, not at the initial stages of puberty. This approach results in less rhGH waste and patient burden compared with the current strategy that recommends rhGH treatment until reaching NAH [27, 62].

However, implementing this strategy has several disadvantages, including the need to revise current guidelines and potential risks of the physician's credibility due to contrasts with explanations and patient expectations at the time of IIGHD diagnosis and start of rhGH treatment. Patients and parents might refuse retesting. Furthermore, if the GH peak is low at mid-puberty, another retest might be required when NAH is reached. Interpreting retest results can be challenging due to factors such as cutoff values, refractory periods, and the effect of BMI [7, 49, 63]. Parents and patients should be informed from the start of rhGH treatment that the IIGHD diagnosis can be revoked in puberty, and re-evaluation of GH secretion will be necessary at some point.

The available evidence suggests that discontinuing rhGH treatment in adolescents with a normal GHST at mid-puberty does not lead to a lower AH compared with continuing treatment [25, 26]. Cavarzere et al conducted a retrospective study in 80 children with IIGHD and found no significant differences in AH between those who were GH sufficient and discontinued rhGH treatment and those who remained GH deficient and restarted treatment until NAH [26]. Similarly, Zucchini et al found that in the 36% of subjects with IIGHD who retested sufficient during mid-puberty, discontinuing rhGH treatment did not negatively impact their growth. Their AH was not statistically different between those who retested deficient and continued treatment [25]. A recent prospective patient preference study conducted by our group showed that in adolescents with IIGHD who tested GH sufficient in mid-puberty, NAH is comparable between those who continued rhGH treatment until NAH and those who stopped treatment at mid-puberty. These findings suggest that in cases of transient IIGHD, rhGH treatment can be safely discontinued in mid-puberty without compromising AH outcomes [64].

In this context, it is also important to discuss the treatment experience from the child's perspective and the impact on parents. Interviews among children and adolescents with GHD aged 8 to <13 years, and parents of children with GHD aged ≥4 to <13 years, found that the treatment burden of childhood-onset GHD was considerable in both children and their parents, with modifiers such as treatment efficacy and duration influencing the severity of the burden [65]. Therefore, stopping treatment earlier could not only improve quality of life for patients and their families, besides significant savings in healthcare costs [66].

##### Retesting at NAH

At NAH, the current routine of retesting aims to determine whether the patient should continue rhGH treatment into adulthood during the transition to adult care. When considering only the studies conducted at NAH in this review, the mean reversal rate is 69.6%. This approach has several advantages, including the ability to assess GH secretion after complete pubertal development and growth completion, which may reduce the risk of unnecessary testing in patients with persistent GHD. It also aligns with current clinical guidelines and provides a clear decision point at the end of pediatric care. However, this approach also has significant disadvantages, including the highest rhGH waste and patient burden, as well as the potential exposure to years of daily or weekly rhGH injections in patients in whom the IIGHD diagnosis could have been rejected years earlier.

#### Cutoff

In this review, the GH cutoff levels at retesting varied widely, ranging from 2.3 to 20 μg/L. The most frequently used cutoff levels were 10 and 8 μg/L. Current guidelines state that in a child with clinical criteria for GHD, a peak GH concentration below 10 μg/L has traditionally been used to support the diagnosis [63], but this cutoff was initiated when GH standards were considerably less purified (1 mg = 2 IU) than current standards (1 mg = 3 IU). This implies that from the time of reaching full standard purification, it would have been logical to change the cutoff for GHST from 10 to 7.6 μg/L. Still, there is no consensus among pediatric endocrinologists, illustrated by the fact that according to the GH Research Society (GRS) guidelines, no specific threshold was agreed upon for a confirmatory diagnosis of GHD. However, the majority of delegates suggested revising the threshold to 7 μg/L, depending on the assay used [16, 63].

The recent GRS Delphi survey concluded that a stimulated GH peak <5 μg/L is consistent with severe GHD in children. In line with this, GH cutoff values of 6 μg/L in Belgium and 5 μg/L in Saudi Arabia were supported as appropriate thresholds during the transition phase during Delphi sessions, with the ITT identified as the preferred diagnostic test [67, 68]. In the Netherlands, a cutoff of 7 μg/L for GH peak has been used. The PES guideline highlights that while peaks below 10 μg/L show some predictive value for adult height SDS, there is no controlled, evidence-based gold standard for this cutoff [62]. This means that lower GH peaks are linked to shorter AH in some studies, but the association is not strong or reliable enough to use as a clear diagnostic rule. No evidence indicates that peak GH values consistently differ across different provocative agents. Due to discrepancies between GH assays, it is recommended that laboratories use harmonized GH assays with the somatropin standard, IRP IS 98/574, 22k rhGH isoform, as per the 2006 and 2011 consensus statements [16, 62, 63].

The 2007 GHD Consensus Workshop compared GH cutoffs in GHSTs for adults and adolescents [27]. For both the ITT and GST, the adult cutoff is defined as a peak GH response of <3 μg/L, whereas during the transition phase, a higher cutoff of <6 μg/L is recommended for the ITT. The AST and GHRH + Arg are frequently applied during the transition phase; however, its outcome is strongly influenced by BMI, which limits its applicability primarily to adolescents with a normal BMI [27, 69, 70]. Unfortunately, since 2022, GHRH is no longer available worldwide.

#### Priming

Nine articles mentioned using priming before diagnosing and/or retesting their patients to minimize the rate of false-positive GH responses. The age when priming was used varied between 9 and 12 years old. Boys were primed with testosterone, with a dosage range between 50 and 125 mg. No boys were primed with estrogen. Girls were primed with ethinyl estradiol, with a dosage range between 20 and 50 µg per day. Vuralli et al [23] showed a significant lower number of insufficient GH peak levels in prepubertal children who were primed compared with those were not primed. Marin et al [17] concluded that both puberty and estrogen administration significantly increase the peak GH response to exercise, arginine, or insulin in healthy subjects. They also noted that the conventional criterion for the peak GH response to exceed 7 µg/L was applicable only to those at Tanner stage 4 or 5, or those who had received estrogen administration [17].

The Pediatric Endocrine Society (PES) guidelines from 2016 stated that sex steroid priming prior to provocative GH testing is suggested to prevent unnecessary rhGH treatment in children with CDGP, specifically in prepubertal boys older than 11 years and prepubertal girls older than 10 years, who have an adult height prognosis within −2 SD of the reference population mean [62]. In a recent Delphi survey by the GRS, 69% of pediatric endocrinologist panelists supported the use of sex steroid priming prior to GH stimulation testing in peripubertal children [71]. Although sex steroid priming may enhance GH response, it remains uncertain whether it fully compensates for the lack of endogenous sex steroids. The GRS guideline published in 2019 states that, while sex steroid priming may improve the specificity of the GH stimulation test, it has not been standardized, the ideal age for priming is undefined, and there is a lack of data on adjusting the cutoff for diagnosing GHD in patients undergoing priming, making its overall efficacy in improving GH provocation testing unclear [63].

#### Other test options to assess the diagnosis of GHD

Alternative approaches to diagnosing GHD have been explored, yet it remains essential to emphasize that the clinical presentation should remain the primary guiding parameter in both diagnosis and treatment decisions. Biochemical markers, such as serum insulin-like growth factor 1 (IGF-1) and insulin-like growth factor binding protein 3, play an important supportive role alongside clinical evaluation and the results of the GHST. Considering that 25-30% of patients with GHD have low-normal IGF-1 concentrations and that low IGF-1 levels can result from other causes such as malnutrition, Ibba et al [32] underscored that IGF-1 measurements alone have poor accuracy in distinguishing between children with GHD and those without, thus necessitating the use of additional clinical and biochemical parameters for accurate diagnosis. In contrast, Fava et al argued that retesting is unnecessary in patients in the transition age with idiopathic GHD and IGF-1 concentrations ≥ 0 SDS, given the low likelihood of persistent GHD in this subgroup, as demonstrated in their study of 97 young adults with childhood-onset GHD aimed at identifying optimal diagnostic cutoffs at adult height [30]. Various authors have attempted to establish a cutoff for IGF-1 or IGF-1 SDS values [7, 30‐32]. It is important to be aware that Postma et al [72] stated that the use of IGF-1 SDS led to increased interlaboratory variation due to the use of different reference value sets.

Two included studies utilized the overnight sleep test [24, 43]. In another study, the authors aimed to capture spontaneous nocturnal GH secretion before a GHST [45]. Participants fasted overnight and remained in a supine position at the outpatient clinic, during the subsequent GHST [45]. Lennartsson et al [73] stated that performing both the arginine–insulin tolerance test and a nocturnal spontaneous GH secretion test reduces the risk of overdiagnosing GHD in short children . Children evaluated with only 1 test were more frequently diagnosed with GHD compared with those evaluated with both tests (48% vs 19%). An overnight 12-hour GH profile could lower the number of children diagnosed with GHD but is labor intensive and a significant burden to patients and their parents and is therefore not used in daily practice in most countries.

Macimorelin, a novel, orally active ghrelin mimetic, stimulates GH secretion and can be used to diagnose adult GHD [74]. In theory, this drug could play a role in diagnosing childhood-onset GHD due to its good tolerability and mild side effect profile [75]. Further investigations are necessary to evaluate its safety and effectiveness in children and adolescents [76].

##### Limitations

This review has several limitations. We aimed to focus on peripubertal retesting, but the scarcity of articles on this specific topic made it challenging to report these findings separately. The risk of bias varied significantly among the included studies: 10 were rated as low risk, 19 as moderate risk, and 2 as serious risk. The 2 studies with serious risk were mainly affected by confounding and missing data, while those with moderate risk had isolated issues such as confounding and participant selection. Low risk studies adhered to robust standards. We did not perform a meta-analysis due to the limited number of randomized controlled trials among the included articles, which limits our ability to quantitatively synthesize the findings.

At the review stage, limitations included incomplete retrieval of identified research and potential reporting bias. In 12 out of 30 studies, it was unclear whether sex steroid priming was performed in prepubertal children at IIGHD diagnosis, and 4 studies did not report the age at diagnosis. These gaps in reporting may have introduced bias and affected the overall findings.

The retrospective nature of most of the included studies posed additional challenges. The global diversity in testing practices complicated the assessment of factors influencing GH response, such as the stimuli used, assay methods and pubertal status at the time of the original IIGHD diagnosis. Finally, most studies included not only patients with IIGHD but also those with multiple pituitary hormone deficiencies, resulting in heterogeneous groups that complicated comparisons. In several papers the exact proportion of patients with IIGHD was only clear after thoroughly examining tables or figures.

## Conclusions

Our findings indicate that retesting for IIGHD can occur at various stages of rhGH treatment, each with distinct advantages and limitations. Specifically, retesting after 1 year of rhGH treatment is recommended for poor responders. In patients with a low clinical likelihood of GHD, retesting may be considered after 1-2 years of treatment. Retesting at mid-puberty is advised for all patients with IIGHD and normal pubertal timing. Clear communication at the start of rhGH treatment can help maintain physician credibility.

In line with PES guidelines, priming before retesting is recommended for prepubertal boys older than 11 years and prepubertal girls older than 10 years; in the Netherlands, lower age thresholds are applied, namely 10 years for boys and 8 years for girls. However, in mid-pubertal adolescents with sufficient endogenous sex steroid production (ie, adequate levels of testosterone or estrogen), priming is generally not necessary. Using the same stimulation test and cutoff level of 7 μg/L as at diagnosis, ensures consistency.

Mid-puberty retesting offers advantages such as reduced false-positive test results due to longer-term endogenous sex steroid exposure, less rhGH waste, and reduced patient burden compared with the current strategy of retesting at NAH. However, this approach carries risks, such as uncertainty in interpreting retest results, and the need for an additional test at NAH if retest results are too low, which increases patient burden.

Future research should aim at optimizing GH retesting protocols to ensure both accurate diagnosis and continuation of effective treatment, while minimizing patient burden and potential loss of efficacy. Studies should explore the long-term outcomes of different retesting strategies and their impact on AH. Additionally, research should focus on improving the accuracy and reliability of GH stimulation tests, especially across varying pubertal stages.

## Data Availability

Some or all datasets generated during and/or analyzed during the current study are not publicly available but are available from the corresponding author on reasonable request.

## References

[1] Mameli C, Guadagni L, Orso M, et al Epidemiology of growth hormone deficiency in children and adolescents: a systematic review. Endocrine. 2024;85(1):91‐98. 38498128 10.1007/s12020-024-03778-4 PMC11246253

[2] Cohen P, Rogol AD, Deal CL, et al Consensus statement on the diagnosis and treatment of children with idiopathic short stature: a summary of the Growth Hormone Research Society, the Lawson Wilkins Pediatric Endocrine Society, and the European Society for Paediatric Endocrinology Workshop. J Clin Endocrinol Metab. 2008;93(11):4210‐4217. 18782877 10.1210/jc.2008-0509

[3] Ortea I, Ruiz-Sánchez I, Cañete R, Caballero-Villarraso J, Cañete MD. Identification of candidate serum biomarkers of childhood-onset growth hormone deficiency using SWATH-MS and feature selection. J Proteomics. 2018;175:105‐113. 29317355 10.1016/j.jprot.2018.01.003

[4] Sizonenko PC, Clayton PE, Cohen P, Hintz RL, Tanaka T, Laron Z. Diagnosis and management of growth hormone deficiency in childhood and adolescence. Growth Horm IGF Res. 2001;11(3):137‐165. 11735230 10.1054/ghir.2001.0203

[5] Yuen KCJ, Johannsson G, Ho KKY, Miller BS, Bergada I, Rogol AD. Diagnosis and testing for growth hormone deficiency across the ages: a global view of the accuracy, caveats, and cut-offs for diagnosis. Endocr Connect. 2023;12(7):e220504. 37052176 10.1530/EC-22-0504 PMC10305501

[6] Bright GM, Morris PA, Rosenfeld RG. When is a positive test for pediatric growth hormone deficiency a true-positive test? Horm Res Paediatr. 2022;94(11-12):399‐405. 10.1159/000521281 34856538

[7] Juul A, Kastrup KW, Pedersen SA, Skakkebaek NE. Growth hormone (GH) provocative retesting of 108 young adults with childhood-onset GH deficiency and the diagnostic value of insulin-like growth factor I (IGF-I) and IGF-binding protein-3. J Clin Endocrinol Metab. 1997;82(4):1195‐1201. 9100596 10.1210/jcem.82.4.3892

[8] Abawi O, Augustijn D, Hoeks SE, de Rijke YB, van den Akker ELT. Impact of body mass index on growth hormone stimulation tests in children and adolescents: a systematic review and meta-analysis. Crit Rev Clin Lab Sci. 2021;58(8):576‐595. 34431447 10.1080/10408363.2021.1956423

[9] Martin LG, Grossman MS, Connor TB, Levitsky LL, Clark JW, Camitta FD. Effect of androgen on growth hormone secretion and growth in boys with short stature. Acta Endocrinol (Copenh). 1979;91(2):201‐212. 463446 10.1530/acta.0.0910201

[10] Yau M, Rapaport R. Growth hormone stimulation testing: to test or not to test? That is one of the questions. Front Endocrinol (Lausanne). 2022;13:902364. 35757429 10.3389/fendo.2022.902364 PMC9218712

[11] Frasier SD . A review of growth hormone stimulation tests in children. Pediatrics. 1974;53(6):929‐937. 4364973

[12] Frasier SD, Hilburn JM, Smith FG. Effect of adolescence on the serum growth hormone response to hypoglycemia. J Pediatr. 1970;77(3):465‐467. 5502100 10.1016/s0022-3476(70)80019-1

[13] Rose SR, Shulman DI, Larsson P, Wakley LR, Wills S, Bakker B. Gender does not influence prepubertal growth velocity during standard growth hormone therapy - analysis of United States KIGS data. J Pediatr Endocrinol Metab. 2005;18(11):1045‐1051. 16459450 10.1515/jpem.2005.18.11.1045

[14] Mauras N . GROWTH HORMONE AND SEX STEROIDS: interactions in puberty. Endocrinol Metab Clin North Am. 2001;30(3):529‐544. 11571929 10.1016/s0889-8529(05)70200-0

[15] Chinoy A, Murray PG. Diagnosis of growth hormone deficiency in the paediatric and transitional age. Best Pract Res Clin Endocrinol Metab. 2016;30(6):737‐747. 27974187 10.1016/j.beem.2016.11.002

[16] Juul A, Bernasconi S, Clayton PE, Kiess W, DeMuinck-Keizer Schrama S. European audit of current practice in diagnosis and treatment of childhood growth hormone deficiency. Horm Res Paediatr. 2002;58(5):233‐241. 10.1159/000066265 12401943

[17] Marin G, Domené HM, Barnes KM, Blackwell BJ, Cassorla FG, Cutler GB. The effects of estrogen priming and puberty on the growth hormone response to standardized treadmill exercise and arginine-insulin in normal girls and boys. J Clin Endocrinol Metab. 1994;79(2):537‐541. 8045974 10.1210/jcem.79.2.8045974

[18] Lazar L, Phillip M. Is sex hormone priming in peripubertal children prior to growth hormone stimulation tests still appropriate? Horm Res Paediatr. 2010;73(4):299‐302. 20215778 10.1159/000284396

[19] Gandrud LM, Wilson DM. Is growth hormone stimulation testing in children still appropriate? Growth Horm IGF Res. 2004;14(3):185‐194. 15125879 10.1016/j.ghir.2003.11.003

[20] Gonc EN, Ozon A, Alikasifoglu A, Kandemir N. Pros of priming in the diagnosis of growth hormone deficiency. J Pediatr Endocrinol Metab. 2011;24(1-2):9‐11. 21528807 10.1515/jpem.2011.076

[21] Clayton PE, Price DA, Shalet SM. Growth hormone state after completion of treatment with growth hormone. Arch Dis Child. 1987;62(3):222‐226. 3566314 10.1136/adc.62.3.222 PMC1778278

[22] Bizzarri C, Pedicelli S, Boscherini B, Bedogni G, Cappa M, Cianfarani S. Early retesting by GHRH + arginine test shows normal GH response in most children with idiopathic GH deficiency. J Endocrinol Invest. 2015;38(4):429‐436. 25376365 10.1007/s40618-014-0205-3

[23] Vuralli D, Gonc EN, Ozon ZA, Alikasifoglu A, Kandemir N. Clinical and laboratory parameters predicting a requirement for the reevaluation of growth hormone status during growth hormone treatment: retesting early in the course of GH treatment. Growth Horm IGF Res. 2017;34:31‐37. 28511077 10.1016/j.ghir.2017.05.003

[24] Cacciari E, Tassoni P, Parisi G, et al Pitfalls in diagnosing impaired growth hormone (GH) secretion: retesting after replacement therapy of 63 patients defined as GH deficient. J Clin Endocrinol Metab. 1992;74(6):1284‐1289. 1592872 10.1210/jcem.74.6.1592872

[25] Zucchini S, Pirazzoli P, Baronio F, et al Effect on adult height of pubertal growth hormone retesting and withdrawal of therapy in patients with previously diagnosed growth hormone deficiency. J Clin Endocrinol Metab. 2006;91(11):4271‐4276. 16912138 10.1210/jc.2006-0383

[26] Cavarzere P, Gaudino R, Sandri M, et al Growth hormone retesting during puberty: a cohort study. Eur J Endocrinol. 2020;182(6):559‐567. 32337961 10.1530/EJE-19-0646

[27] Ho KKY . Consensus guidelines for the diagnosis and treatment of adults with GH deficiency II: a statement of the GH Research Society in association with the European Society for Pediatric Endocrinology, Lawson Wilkins Society, European Society of Endocrinology, Japan Endocrine Society, and Endocrine Society of Australia. Eur J Endocrinol. 2007;157(6):695‐700. 18057375 10.1530/EJE-07-0631

[28] Meazza C, Gertosio C, Pagani S, et al Is retesting in growth hormone deficient children really useful? Minerva Endocrinol (Torino). 2017;42(4):325‐330. 10.23736/S0391-1977.16.02510-4 27304071

[29] Tauber M, Moulin P, Pienkowski C, Jouret B, Rochiccioli P. Growth hormone (GH) retesting and auxological data in 131 GH-deficient patients after completion of treatment. J Clin Endocrinol Metab. 1997;82(2):352‐356. 9024217 10.1210/jcem.82.2.3726

[30] Fava D, Guglielmi D, Pepino C, et al Accuracy of glucagon testing across transition in young adults with childhood-onset GH deficiency. J Clin Endocrinol Metab. 2024;110(1):78‐90. 38913686 10.1210/clinem/dgae408 PMC11651695

[31] Darendeliler F, Spinu I, Bas F, et al Reevaluation of growth hormone deficiency during and after growth hormone (GH) treatment: diagnostic value of GH tests and IGF-I and IGFBP-3 measurements. J Pediatr Endocrinol Metab. 2004;17(7):1007‐1012. 15301049 10.1515/jpem.2004.17.7.1007

[32] Ibba A, Corrias F, Guzzetti C, et al IGF1 for the diagnosis of growth hormone deficiency in children and adolescents: a reappraisal. Endocr Connect. 2020;9(11):1095‐1102. 33112822 10.1530/EC-20-0347 PMC7774770

[33] Vliegenthart J, Wols DF, Wit JM, et al Supplementary materials for “Growth Hormone Retesting for Idiopathic Isolated Growth Hormone Deficiency During and After Puberty: A Systematic Review, Journal of the Endocrine Society, 2026”. DataverseNL V1. Date of deposit 6 May 2026. 10.34894/R6Q27B. PMC13247589 42272566

[34] Bramer WM, Giustini D, de Jonge GB, Holland L, Bekhuis T. De-duplication of database search results for systematic reviews in EndNote. J Med Libr Assoc. 2016;104(3):240‐243. 27366130 10.3163/1536-5050.104.3.014 PMC4915647

[35] Liberati A, Altman DG, Tetzlaff J, et al The PRISMA statement for reporting systematic reviews and meta-analyses of studies that evaluate healthcare interventions: explanation and elaboration. BMJ. 2009;339:b2700. 19622552 10.1136/bmj.b2700 PMC2714672

[36] Page MJ, McKenzie JE, Bossuyt PM, et al The PRISMA 2020 statement: an updated guideline for reporting systematic reviews. BMJ. 2021;372:n71. 33782057 10.1136/bmj.n71 PMC8005924

[37] Sterne JA, Hernán MA, Reeves BC, et al ROBINS-I: a tool for assessing risk of bias in non-randomised studies of interventions. BMJ. 2016;355:i4919. 27733354 10.1136/bmj.i4919 PMC5062054

[38] McGuinness LA, Higgins JPT. Risk-of-bias VISualization (robvis): an R package and Shiny web app for visualizing risk-of-bias assessments. Res Synth Methods. 2021;12(1):55‐61. 32336025 10.1002/jrsm.1411

[39] Ahmid M, Fisher V, Graveling AJ, et al An audit of the management of childhood-onset growth hormone deficiency during young adulthood in Scotland. Int J Pediatr Endocrinol. 2016;2016(1):6. 26985190 10.1186/s13633-016-0024-8 PMC4793498

[40] Aimaretti G, Baffoni C, Bellone S, et al Retesting young adults with childhood-onset growth hormone (GH) deficiency with GH-releasing-hormone-plus-arginine Test. J Clin Endocrinol Metab. 2000;85(10):3693‐3699. 11061526 10.1210/jcem.85.10.6858

[41] Berberoǧlu M, Siklar Z, Darendeliler F, et al Evaluation of permanent growth hormone deficiency (GHD) in young adults with childhood onset GHD: a multicenter study. J Clin Res Pediatr Endocrinol. 2011;1(1):30‐37. 10.4008/jcrpe.v1i1.7 PMC3005632 21318062

[42] Bonfig W, Bechtold S, Bachmann S, et al Reassessment of the optimal growth hormone cut-off level in insulin tolerance testing for growth hormone secretion in patients with childhood-onset growth hormone deficiency during transition to adulthood. Pediatr Endocrinol Metab. 2008;21(11):1049‐1056. 10.1515/jpem.2008.21.11.1049 19189699

[43] Cacciari E, Tassoni P, Cicognani A, et al Value and limits of pharmacological and physiological tests to diagnose growth hormone (GH) deficiency and predict therapy response: first and second retesting during replacement therapy of patients defined as GH deficient. J Clin Endocrinol Metab. 1994;79(6):1663‐1669. 7989472 10.1210/jcem.79.6.7989472

[44] Deillon E, Hauschild M, Faouzi M, et al Natural history of growth hormone deficiency in a pediatric cohort. Horm Res Paediatr. 2015;83(4):252‐261. 25676059 10.1159/000369392

[45] Dreismann L, Schweizer R, Blumenstock G, Weber K, Binder G. Evaluation of the GHRH-arginine retest for young adolescents with childhood-onset GH deficiency. Growth Horm IGF Res. 2016;27:28‐32. 26874855 10.1016/j.ghir.2016.02.001

[46] Gelwane G, Garel C, Chevenne D, et al Subnormal serum insulin-like growth factor-I levels in young adults with childhood-onset nonacquired growth hormone (GH) deficiency who recover normal GH secretion may indicate less severe but persistent pituitary failure. J Clin Endocrinol Metab. 2007;92(10):3788‐3795. 17666477 10.1210/jc.2007-1003

[47] Gökşen D, Çoker M, Özkayin N, Darcan Ş. Evaluation of growth hormone secretion after completion of therapy. Pediatr Int. 2001;43(2):137‐140. 11285064 10.1046/j.1442-200x.2001.01363.x

[48] Longobardi S, Merola B, Pivonello R, et al Reevaluation of growth hormone (GH) secretion in 69 adults diagnosed as GH-deficient patients during childhood. J Clin Endocrinol Metab. 1996;81(3):1244‐1247. 8772606 10.1210/jcem.81.3.8772606

[49] Maghnie M, Strigazzi C, Tinelli C, et al Growth hormone (GH) deficiency (GHD) of childhood onset: reassessment of GH status and evaluation of the predictive criteria for permanent GHD in young adults. J Clin Endocrinol Metab. 1999;84(4):1324‐1328. 10199773 10.1210/jcem.84.4.5614

[50] Maghnie M, Aimaretti G, Bellone S, et al Diagnosis of GH deficiency in the transition period: accuracy of insulin tolerance test and insulin-like growth factor-I measurement. Eur J Endocrinol. 2005;152(4):589‐596. 15817915 10.1530/eje.1.01873

[51] Nicolson A, Toogood AA, Rahim A, Shalet SM. The prevalence of severe growth hormone deficiency in adults who received growth hormone replacement in childhood. Clin Endocrinol (Oxf). 1996;44(3):311‐316. 8729528 10.1046/j.1365-2265.1996.671492.x

[52] Pauwels C, Souberbielle JC, Prévot-Saucet C, Brauner R. The capacity to increase GH secretion at puberty reflects the severity of GH deficiency. Horm Res. 1992;38(3-4):140‐144. 1306845 10.1159/000182529

[53] Penta L, Cofini M, Lucchetti L, et al Growth hormone (GH) therapy during the transition period: should we think about early retesting in patients with idiopathic and isolated GH deficiency? Int J Environ Res Public Health. 2019;16(3):307. 30678118 10.3390/ijerph16030307 PMC6388362

[54] Quigley CA, Zagar AJ, Liu CC, et al United States multicenter study of factors predicting the persistence of GH deficiency during the transition period between childhood and adulthood. Int J Pediatr Endocrinol. 2013;2013(1):6. 23406437 10.1186/1687-9856-2013-6 PMC3605263

[55] Smyczyńska J, Stawerska R, Lewiński A, Hilczer M. Incidence and predictors of persistent growth hormone deficiency (GHD) in patients with isolated, childhood-onset GHD. Endokrynol Pol. 2014;65(5):334‐341. 25301482 10.5603/EP.2014.0046

[56] Smyczyńska J, Hilczer M, Smyczyńska U, Lewiński A, Stawerska R. Transient isolated, idiopathic growth hormone deficiency—a self-limiting pediatric disease with male predominance or a diagnosis based on uncertain criteria? Lesson from 20 years' real-world experience with retesting at one center. Int J Mol Sci. 2024;25(11):5739. 38891927 10.3390/ijms25115739 PMC11171613

[57] Thomas M, Massa G, Maes M, et al Growth hormone (GH) secretion in patients with childhood-onset GH deficiency: retesting after one year of therapy and at final height. Horm Res Paediatr. 2003;59(1):7‐15. 10.1159/000067936 12566729

[58] Wacharasindhu S, Cotterill AM, Camacho-Hübner C, Besser GM, Savage MO. Normal growth hormone secretion in growth hormone insufficient children retested after completion of linear growth. Clin Endocrinol (Oxf). 1996;45(5):553‐556. 8977751 10.1046/j.1365-2265.1996.00850.x

[59] Mrcpch SW, Aroonparkmongkol Bsc S, Sahakitrungrueng T, Supornsilchai V. Growth hormone (GH) retesting and final adult height in childhood-onset GH deficiency (CO-GHD): experiences from King Chulalongkorn Memorial Hospital, Thailand. J Med Assoc Thai. 2015;98(6):542‐548. 26219157

[60] Cuboni D, Caputo M, Ghigo E, Aimaretti G, Gasco V. Once upon a time: the glucagon stimulation test in diagnosing adult GH deficiency. J Endocrinol Invest. 2024;47(7):1621‐1631. 38461479 10.1007/s40618-024-02322-5 PMC11196325

[61] Kargi AY, Merriam GR. Testing for growth hormone deficiency in adults. Curr Opin Endocrinol Diabetes Obes. 2012;19(4):300‐305. 22596248 10.1097/MED.0b013e32835430da

[62] Grimberg A, DiVall SA, Polychronakos C, et al Guidelines for growth hormone and insulin-like growth factor-I treatment in children and adolescents: growth hormone deficiency, idiopathic short stature, and primary insulin-like growth factor-I deficiency. Horm Res Paediatr. 2016;86(6):361‐397. 27884013 10.1159/000452150

[63] Collett-Solberg PF, Ambler G, Backeljauw PF, et al Diagnosis, genetics, and therapy of short stature in children: a growth hormone research society international perspective. Horm Res Paediatr. 2019;92(1):1‐14. 10.1159/000502231 PMC6979443 31514194

[64] Vliegenthart J, Wit JM, Bakker B, et al Growth hormone withdrawal in mid-puberty: no impact on near adult height in adolescents with transient idiopathic GHD. J Clin Endocrinol Metab. Published online November 15, 2025. Doi:10.1210/clinem/dgaf626 PMC13099207 41239863

[65] Brod M, Højbjerre L, Alolga SL, Beck JF, Wilkinson L, Rasmussen MH. Understanding treatment burden for children treated for growth hormone deficiency. Patient. 2017;10(5):653‐666. 28386679 10.1007/s40271-017-0237-9 PMC5605605

[66] Brettell E, Högler W, Woolley R, et al The growth hormone deficiency (GHD) reversal trial: effect on final height of discontinuation versus continuation of growth hormone treatment in pubertal children with isolated GHD—a non-inferiority randomised controlled trial (RCT). Trials. 2023;24(1):548. 37605233 10.1186/s13063-023-07562-z PMC10440873

[67] Staels W, De Schepper J, Becker M, et al Policy for transitioning childhood-onset growth hormone deficiency from pediatric to adult endocrine care in Belgium. Front Endocrinol (Lausanne). 2024;15:1459998. 39415786 10.3389/fendo.2024.1459998 PMC11482521

[68] AlMutair A, Alsagheir A, AlShammary A, et al Management of childhood-onset growth hormone deficiency in patients transitioning from pediatric to adult care: a review of the literature and consensus report from a panel of experts in Saudi Arabia. Int J Pediatr Adolesc Med. 2023;10(2):21‐30.

[69] Gasco V, Cuboni D, Varaldo E, et al GHRH+arginine test and body mass index: do we need to review diagnostic criteria for GH deficiency? J Endocrinol Invest. 2023;46(10):2175‐2183. 37062055 10.1007/s40618-023-02081-9 PMC10514141

[70] Perotti M, Perra S, Saluzzi A, Grassi G, Pincelli A. Body fat mass is a strong and negative predictor of peak stimulated growth hormone and bone mineral density in healthy adolescents during transition period. Horm Metab Res. 2013;45(10):748‐753. 23913118 10.1055/s-0033-1347243

[71] Arlien-Søborg MC, Radovick S, Boguszewski MCS, et al Consensus and controversies about diagnosing GH deficiency: a Delphi survey by the GH research society. Pituitary. 2025;28(3):57. 40335774 10.1007/s11102-025-01526-z

[72] Postma MR, van Beek AP, van der Klauw MM, Lentjes EGWM, Muller Kobold AC. IGF-1 as screening tool for acromegaly and adult-onset growth hormone deficiency in the Netherlands. Clin Endocrinol (Oxf). 2024;100(3):260‐268. 38044875 10.1111/cen.15000

[73] Lennartsson O, Nilsson O, Lodefalk M. Discordance between stimulated and spontaneous growth hormone levels in short children is dependent on cut-off level and partly explained by refractoriness. Front Endocrinol (Lausanne). 2020;11:584906. 33281744 10.3389/fendo.2020.584906 PMC7705110

[74] Garcia JM, Swerdloff R, Wang C, et al Macimorelin (AEZS-130)-stimulated growth hormone (GH) test: validation of a novel oral stimulation test for the diagnosis of adult GH deficiency. J Clin Endocrinol Metab. 2013;98(6):2422‐2429. 23559086 10.1210/jc.2013-1157 PMC4207947

[75] Csákváry V, Ammer N, Bagci EB, et al Safety, tolerability, pharmacokinetics, and pharmacodynamics of Macimorelin in children with suspected growth hormone deficiency: an open-label, group comparison, dose-escalation trial. Horm Res Paediatr. 2022;94(7-8):239‐250. 10.1159/000519232 34438400

[76] Henry RK . Macimorelin acetate for the diagnosis of childhood-onset growth hormone deficiency. Eur Endocrinol. 2022;18(2):84. 10.17925/EE.2022.18.2.84 PMC9835807 36694890

